# Non-Albicans *Candida* Peritonitis in Peritoneal Dialysis: Species Distribution, Management, and Outcomes—A Systematic Case-Based Review

**DOI:** 10.3390/idr18030041

**Published:** 2026-04-27

**Authors:** John Dotis, Athina Papadopoulou, Maria Fourikou, Marianna Papakonstantinou, Ioustini Kalaitzopoulou, Charalampos Antachopoulos

**Affiliations:** 1 Third Department of Pediatrics, Hippokration Hospital, Aristotle University of Thessaloniki, 54642 Thessaloniki, Greece; mfour@auth.gr (M.F.); marianpapak@gmail.com (M.P.); ioustinek@gmail.com (I.K.); antachop@auth.gr (C.A.); 2 First Department of Pediatrics, Hippokration Hospital, Aristotle University of Thessaloniki, 54642 Thessaloniki, Greece; athinapap80@yahoo.gr

**Keywords:** peritoneal dialysis, fungal peritonitis, non-albicans *Candida*, antifungal therapy, catheter removal, systematic review

## Abstract

Background/Objectives: Fungal peritonitis is a severe complication of peritoneal dialysis (PD) associated with catheter removal, technique failure, and increased mortality. Although *Candida albicans* was traditionally the predominant pathogen, non-albicans *Candida* (NAC) species are increasingly reported. This review summarizes the epidemiology and outcomes of PD-associated NAC peritonitis. Methods: A systematic review was performed following PRISMA guidelines. PubMed/MEDLINE, Scopus, and Google Scholar were searched (January 1990–March 2026) for NAC peritonitis studies. Case reports and series with species-level identification were included. Results: 31 studies met the inclusion criteria, comprising 25 individual case reports and 6 case series, totaling 89 NAC isolates. *Candida parapsilosis* was the most frequently reported species (*n* = 50), followed by *Candida tropicalis* (*n* = 15). Other pathogens included *Candida glabrata*, *Candida guilliermondii,* and several rare NAC species. Fluconazole was the most commonly used initial antifungal therapy. Catheter removal was performed in most cases, with the majority of patients requiring transition to hemodialysis. Overall mortality was 20% among individual case reports vs. 24% across case series. Species-specific differences were observed: *C. parapsilosis* and *C. guilliermondii* were generally associated with favorable outcomes, whereas infections involving *C. glabrata* and other emerging NAC species more frequently required treatment escalation and were linked to poorer outcomes. Conclusions: NAC species are an important cause of fungal peritonitis in PD patients and show considerable heterogeneity in clinical outcomes and antifungal susceptibility. Early species-level identification and prompt catheter removal remain essential for optimal management.

## 1. Introduction

Peritoneal dialysis (PD)–related peritonitis remains one of the most important complications associated with PD therapy and is a major cause of morbidity, technique failure, and transition to hemodialysis (HD). Despite improvements in dialysis technology, infection prevention strategies, and patient training, infectious complications continue to affect long-term PD outcomes. Current recommendations from the International Society for Peritoneal Dialysis (ISPD) emphasize the importance of early diagnosis and prompt treatment to improve clinical outcomes [1].

Fungal peritonitis represents a relatively uncommon but particularly severe form of PD-related infection [2]. Compared with bacterial peritonitis, fungal infections are associated with significantly higher rates of catheter removal, technique failure, and mortality [3,4]. Consequently, fungal peritonitis remains a serious clinical entity requiring rapid recognition and aggressive management.

Historically, *Candida albicans* has been considered the predominant pathogen in intra-abdominal candidiasis. However, infections caused by non-albicans *Candida* (NAC) species have been increasingly reported in recent years [4]. These organisms include *Candida parapsilosis*, *Candida tropicalis*, *Candida glabrata,* and *Candida guilliermondii*, among others. Importantly, NAC species may differ from *C. albicans* in epidemiology, virulence characteristics, and antifungal susceptibility patterns, with some species demonstrating reduced susceptibility to commonly used azole antifungal agents [5].

In patients undergoing PD, several factors have been associated with an increased risk of fungal peritonitis. Prior bacterial peritonitis and prolonged exposure to broad-spectrum antibiotics are among the most consistently reported risk factors, as they may disrupt the normal microbial balance and facilitate fungal colonization of the peritoneal cavity [2,4]. In addition, colonization of PD catheters and the ability of certain *Candida* species to form biofilms on synthetic surfaces may contribute to the development of infection.

Evidence regarding NAC peritonitis in PD remains limited and fragmented. Most published data derive from isolated case reports or small case series describing individual episodes of infection [6,7,8]. Consequently, the epidemiology, species distribution, and clinical outcomes of NAC peritonitis remain incompletely characterized.

Given these limitations, a comprehensive evaluation of the available evidence is warranted. Therefore, the objective of the present systematic review was to compile and analyze all published cases of PD-associated peritonitis caused by NAC species. Particular emphasis was placed on species distribution, patient characteristics, therapeutic strategies, and reported mortality outcomes.

## 2. Materials and Methods

### 2.1. Study Design and Reporting Framework

This systematic review was conducted in accordance with the Preferred Reporting Items for Systematic Reviews and Meta-Analyses (PRISMA) 2020 guidelines for transparent reporting of systematic evidence syntheses [9]. The review protocol was prospectively registered in the International Prospective Register of Systematic Reviews (PROSPERO; registration number CRD420261329285).

### 2.2. Eligibility Criteria and Definitions

Eligible studies included case reports and case series describing patients undergoing PD who developed microbiologically confirmed peritonitis caused by NAC species. Peritonitis was defined according to established clinical criteria, requiring the presence of compatible symptoms (e.g., abdominal pain, fever, nausea, or cloudy effluent) combined with a peritoneal effluent white blood cell count ≥100 cells/μL with ≥50% polymorphonuclear leukocytes. Microbiological confirmation required isolation of the NAC species from peritoneal fluid culture using conventional culture-based methods, with species-level identification performed by biochemical or phenotypic identification systems. When available, additional confirmatory techniques, such as matrix-assisted laser desorption/ionization time-of-flight mass spectrometry (MALDI-TOF MS) or molecular sequencing methods, were also recorded. Only cases with species-level identification were included. Reports limited to *C. albicans* were excluded. In mixed fungal or polymicrobial infections, cases were included only when outcome data could be clearly attributed to the NAC isolate.

Primary fungal peritonitis was defined as peritonitis caused by NAC occurring in the absence of a preceding episode of bacterial peritonitis. Secondary fungal peritonitis was defined as an episode occurring within 30 days following exposure to antimicrobial therapy for bacterial peritonitis or concomitantly with a documented bacterial infection. Early-onset peritonitis was defined as the first episode occurring within 6 months of PD initiation, whereas late-onset peritonitis was defined as occurring after 6 months.

Cases without microbiological confirmation of NAC species, without extractable individual patient-level outcome data, or not clearly associated with PD therapy were excluded from the analysis.

### 2.3. Information Sources and Search Strategy

A comprehensive literature search was conducted in PubMed/MEDLINE, Scopus, and Google Scholar from 1 January 1990 through 15 March 2026, in accordance with the predefined protocol registered in PROSPERO. The search strategy combined controlled vocabulary and free-text terms related to PD, peritonitis and NAC species using Boolean operators, including (“peritoneal dialysis” OR “PD” OR “CAPD” OR “automated peritoneal dialysis”) AND (“peritonitis”) AND (“non-albicans *Candida*” OR “*Candida parapsilosis*” OR “*Candida tropicalis*” OR “*Candida glabrata*” OR “*Candida guilliermondii*” OR “*Candida lusitaniae*” OR “*Candida haemulonii*” OR “*Candida nivariensis*” OR “*Candida famata*” OR other non-albicans *Candida* species names). In Google Scholar, the first 300 results ranked by relevance were screened as prespecified. Backward reference list screening and forward citation tracking were additionally performed. No language restrictions were applied; publications in languages other than English were translated using publicly available tools (e.g., Google Translate) and subsequently reviewed by the authors to ensure accuracy. When necessary, key clinical and methodological information was cross-checked against the original text to ensure consistency in data extraction.

### 2.4. Study Selection

All potentially relevant records were screened in two stages. Initially, titles and abstracts were independently assessed by two reviewers (J.D., A.P.) to determine eligibility. Full texts of studies meeting the inclusion criteria were subsequently retrieved and independently evaluated (J.D., A.P., M.F.) Reference lists of all included articles were manually screened to ensure comprehensive case identification. Discrepancies were resolved through discussion and consensus among the review team (J.D., A.P., M.F., M.P., I.K., and C.A.).

### 2.5. Data Extraction

Data were extracted independently by two reviewers (J.D., A.P.) using a predefined standardized data collection form. Extracted variables included year of publication, country, demographic characteristics (age and sex), underlying comorbidities, PD modality and vintage, prior episodes of peritonitis, identified NAC species, and method of microbiological identification. Treatment-related variables included the antifungal agent, route and duration of therapy, timing of peritoneal catheter removal, and, when applicable, the switch to HD. The primary outcome (mortality) and secondary outcomes (technique failure and catheter removal) were recorded as reported in each study. When data were incomplete or unclear, only extractable and explicitly reported variables were included in the analysis. Discrepancies in data extraction were resolved through discussion and consensus between authors. Publications were categorized according to study design as single-patient case reports or multi-patient case series. Analyses were primarily stratified by publication type to account for inherent differences in reporting structure and data completeness. Species-level summaries were subsequently derived from pooled individual cases across both publication types.

### 2.6. Risk of Bias Assessment

Risk of bias was assessed using the Joanna Briggs Institute (JBI) critical appraisal tools for case reports and case series. Two investigators (J.D. and A.P.) independently evaluated study quality, with disagreements resolved through discussion and consensus. The assessment considered key methodological domains, including clarity of patient description, diagnostic ascertainment, completeness of clinical data, and transparency of outcome reporting. The study-level appraisal is presented in Supplementary Tables S1 and S2. Overall, most studies were judged to be of moderate to high methodological quality.

### 2.7. Statistical Analysis

Extracted individual patient-level data were compiled into a master database using Microsoft Excel (Microsoft Corp., Redmond, WA, USA) and subsequently analyzed using GraphPad Prism version 8 (GraphPad Software Inc., San Diego, CA, USA). Categorical variables were summarized as frequencies and percentages, while continuous variables were reported as mean ± standard deviation or median (range), as appropriate. Species-specific mortality rates were calculated as proportions of deaths among reported cases for each NAC species.

## 3. Results

**Figure 1:**
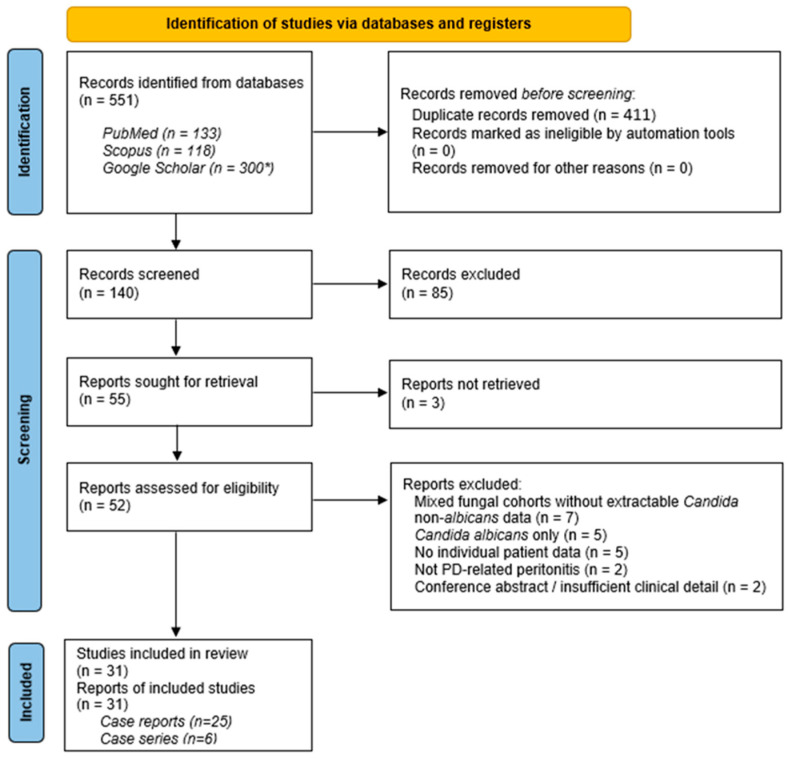
PRISMA 2020 flow diagram of the study selection process. * Only the first 300 records retrieved from Google Scholar were screened.

The selection process according to the PRISMA 2020 framework is illustrated in Figure 1. In total, 551 records were initially identified through database searches (PubMed, Scopus, and Google Scholar). After removal of duplicates, 140 records were screened by title and abstract, of which 85 were excluded. Fifty-five reports were sought for retrieval, and 52 were assessed for eligibility.

Ultimately, 31 studies met the inclusion criteria, comprising 25 individual case reports [10,11,12,13,14,15,16,17,18,19,20,21,22,23,24,25,26,27,28,29,30,31,32,33,34] (Table 1) and six case series [6,7,8,35,36,37] describing 64 episodes of NAC peritonitis in 63 patients (Table 2). In total, 89 episodes were identified across 88 patients, as one patient experienced two episodes [7].

The individual case reports originated from multiple geographic regions, including Europe, Asia, and North America. Most patients were adults receiving continuous ambulatory PD (CAPD), although one neonatal case undergoing acute PD [19] and one patient receiving automated PD (APD) [30] were also described. Catheter removal was performed in most individual cases (92%), and most patients were subsequently transitioned to HD (84%). Detailed analysis of available data confirmed HD use in 23/25 patients (92%; 100% among cases with reported data), while reinstitution of PD was documented in only 3/22 cases (14%). Overall mortality among the reported individual cases was 20% (Table 3).

Fluconazole was the most frequently used initial antifungal therapy in the individual reports (60%), followed by amphotericin B (28%) and echinocandins (12%). In several cases, treatment escalation or modification was required because of clinical non-response or suspected antifungal resistance.

The six-case series included 64 reported episodes of NAC peritonitis in 63 patients. In all studies, catheter removal was performed (100%), and most patients were transitioned to HD. In most instances, patients remained on HD permanently, while only a few resumed PD after delayed reinsertion of a new Tenckhoff catheter. However, detailed data regarding dialysis modality following the acute episode were limited and inconsistently reported across studies. Mortality rates in the individual series ranged from 0% to 60%, with an overall mortality of 24%. Fluconazole was the predominant initial antifungal therapy (72%), whereas amphotericin B and echinocandins were used less frequently (8% and 4%, respectively).

Across all included studies, 89 NAC isolates were identified (Table 4).

The most frequently reported species was *C. parapsilosis (n* = 50), followed by *C. tropicalis* (*n* = 15). Less commonly reported species included *C. glabrata* (*n* = 4) and *C. guilliermondii* (*n* = 4), whereas several rare species were each represented by single reports, including rare NAC species. Species-specific mortality patterns varied across the included reports. Among individual case reports, deaths were observed in infections caused by *C. famata* (2/2), *C. lusitaniae* (1/2), *C. haemulonii* (1/1), *C. nivariensis* (1/1), and *C. parapsilosis* (1/5). In the case series, mortality occurred in 3 of 5 episodes of *C. tropicalis* peritonitis and in 5 of 31 episodes of *C. parapsilosis* peritonitis for which outcome data were available.

Evaluation of the individual case reports revealed notable species-specific clinical patterns (Table 5).

Infections caused by *C. parapsilosis* were generally associated with favorable outcomes, with universal catheter removal. Similarly, infections due to *C. guilliermondii* showed consistently good outcomes in all reported cases. In contrast, infections caused by *C. glabrata* were frequently associated with prior antibiotic exposure (100%) and occasional polymicrobial infection (33%), often requiring escalation of antifungal therapy to echinocandins.

Finally, several reports involving emerging species suggested potential antifungal susceptibility challenges. Cases caused by *C. glabrata* frequently required treatment modification because of reduced susceptibility to fluconazole. In contrast, rare species, such as *C. nivariensis* and *C. haemulonii*, were associated with severe clinical courses or suspected antifungal resistance in the original reports. Overall, these findings illustrate the considerable heterogeneity in species distribution, clinical presentation, and therapeutic response among cases of NAC peritonitis in patients receiving PD.

## 4. Discussion

This systematic review provides an integrated overview of NAC peritonitis in patients undergoing PD. The available evidence indicates substantial heterogeneity among NAC species with respect to species distribution, antifungal susceptibility, and clinical outcomes. Specifically, *C. parapsilosis* and *C. tropicalis* were the most frequently reported pathogens. In contrast, infections caused by *C. glabrata* and several emerging NAC species were more often associated with reduced susceptibility to azole antifungal agents. These findings highlight the clinical importance of early species-level identification to guide targeted antifungal therapy and support timely management decisions in PD-associated fungal peritonitis.

One of the most notable findings of this analysis was the predominance of *C. parapsilosis*, which accounted for more than half of the identified NAC isolates. This observation is consistent with previous reports indicating that *C. parapsilosis* is frequently associated with catheter-related infections and the formation of biofilms on indwelling medical devices, including PD catheters. Its ability to adhere to artificial surfaces and produce biofilms may facilitate colonization of the PD system and contribute to subsequent infection [37,38,39]. Similar species distributions have been reported in regional PD cohorts, in which *C. parapsilosis* emerged as one of the most common fungal pathogens after *C. albicans* [7,8,39].

Despite its frequent occurrence, infections caused by *C. parapsilosis* in the present review were generally associated with favorable clinical outcomes, with only one death reported among individual case reports. This observation may reflect the relatively preserved susceptibility of this species to azole antifungal agents, particularly fluconazole, which was also the most common initial antifungal therapy in the included cases [3]. Nevertheless, *C. parapsilosis* has been reported to demonstrate reduced susceptibility to echinocandins compared with other NAC species, a factor that may influence therapeutic decision-making in severe infections [38].

The second most frequently reported pathogen was *C. tropicalis*, a species previously associated with invasive candidiasis in immunocompromised individuals and in patients exposed to broad-spectrum antibiotic therapy. Although the number of cases identified in the present review was relatively small, clinical outcomes were generally favorable when prompt antifungal treatment and catheter removal were implemented. Previous observational studies have similarly suggested that early therapeutic intervention combined with appropriate antifungal therapy can significantly improve outcomes in fungal PD peritonitis [36,39,40].

In contrast, infections caused by *C. glabrata* appeared to follow a more complicated clinical course. All reported cases were associated with prior antibiotic therapy, with some requiring escalation of antifungal treatment to echinocandins due to suspected or documented azole resistance. This observation is consistent with the well-documented reduced susceptibility of *C. glabrata* to fluconazole and other azole agents, which has been increasingly recognized in invasive candidiasis [5]. The relatively higher mortality observed in these cases further suggests that infections caused by this species may present greater therapeutic challenges.

Another notable observation from this review is the broad diversity of rare or emerging NAC species implicated in PD-associated peritonitis. Several unusual organisms, including *C. haemulonii*, *C. nivariensis*, *Candida pelliculosa* (*Wickerhamomyces anomalus*), and *Candida subhashii*, were identified in isolated reports. The increasing recognition of these organisms likely reflects advances in diagnostic technologies, particularly the implementation of MALDI-TOF mass spectrometry and molecular identification techniques in clinical microbiology laboratories [23,28]. Importantly, some of these species have been associated with reduced susceptibility to commonly used antifungal agents, raising concerns regarding treatment selection and potential clinical outcomes [2,5].

In line with current international recommendations, peritoneal catheter removal was performed in the vast majority of cases included in this review. Early removal of the PD catheter has long been considered a key component of management in fungal peritonitis, as catheter retention is associated with persistent infection and poorer clinical outcomes. The ISPD guidelines strongly recommend prompt catheter removal once fungal peritonitis is diagnosed, followed by systemic antifungal therapy and transition to HD [1]. The high rate of catheter removal observed in the present study likely reflects adherence to these widely accepted management recommendations.

Fluconazole was the most frequently administered antifungal agent across both individual case reports and case series. This likely reflects its broad availability, favorable pharmacokinetic properties, and well-established role in the treatment of susceptible *Candida* infections [3]. However, the need for treatment modification in several cases emphasizes the importance of species-level identification and antifungal susceptibility testing whenever feasible. Emerging resistance patterns among NAC species may necessitate the use of alternative antifungal agents, including echinocandins or lipid formulations of amphotericin B [5].

Overall mortality across the studies included in the present review ranged from approximately 20% in individual case reports to 24% in case series. These findings are generally consistent with previously reported mortality rates in fungal PD peritonitis, which ranged between 15% and 40% in earlier observational studies [36,39,40]. Although mortality remains considerable, outcomes appear to vary according to the infecting species, patient characteristics, and the timing of therapeutic interventions.

Several limitations of the present review should be acknowledged. First, the available evidence consists predominantly of single case reports and small case series, limiting the ability to perform robust statistical comparisons across species. Second, important clinical variables, including antifungal susceptibility data, timing of catheter removal, modality resumption, and detailed patient comorbidities, were inconsistently reported across studies. In addition, clinically relevant parameters, such as the duration of peritoneal dialysis and prior episodes of peritonitis and their treatments, were not consistently reported and could not be systematically analyzed. Third, publication bias cannot be excluded, as unusual or severe cases may be more likely to be reported in the literature. Consequently, the findings of this review should primarily be interpreted as descriptive and hypothesis-generating.

Despite these limitations, this study provides a comprehensive overview of the spectrum of NAC species responsible for PD-associated peritonitis and highlights potential differences in clinical presentation, management, and outcomes. These findings underscore the clinical relevance of species-level identification and support the importance of individualized antifungal management in PD-associated fungal infections. Continued multicenter surveillance and systematic case reporting will be important to improve understanding of species-specific epidemiology and guide appropriate antifungal management.

## 5. Conclusions

NAC species are increasingly recognized as a cause of PD–associated fungal peritonitis and exhibit substantial heterogeneity in species distribution, antifungal susceptibility, and clinical outcomes. In this systematic synthesis of published cases, *C. parapsilosis* and *C. tropicalis* emerged as the most frequently reported pathogens. In contrast, infections due to *C. glabrata* and other emerging NAC species were more often associated with therapeutic challenges. Early species-level identification, prompt initiation of appropriate antifungal therapy, and timely catheter removal remain key components of optimal management. Future prospective studies and multicenter surveillance are needed to better define species-specific risk factors, resistance patterns, and optimal treatment strategies for NAC peritonitis in patients receiving PD.

## Figures and Tables

**Table 1:** Individual case reports of NAC peritonitis in patients receiving PD.

Reference/Year	Country	Age/Sex	PD Modality	Candida Species	Initial Antifungal Therapy	Catheter Removed	Outcome
[10]/1994	Spain	76/M	CAPD	*Candida famata*	Fluconazole	No	Death
[11]/1997	Japan	41/F	CAPD	*Candida glabrata*	Fluconazole	Yes	Survived
[12]/1999	UK	50/M	CAPD	*Candida parapsilosis*	Fluconazole + flucytosine	Yes	Survived
[13]/2002	Turkey	28/F	CAPD	*Candida lusitaniae*	Fluconazole	Yes	Survived
[14]/2004	Saudi Arabia	69/F	CAPD	*Candida lusitaniae*	None before death	Yes	Death
[15]/2006	UK	35/M	CAPD	*Candida famata*	Fluconazole + amphotericin B	Yes	Death
[16]/2009	Canada	69/M	CAPD	*Candida subhashii*	Fluconazole	Yes	Survived
[17]/2009	Turkey	41/M	CAPD	*Candida sake*	Fluconazole	Yes	Survived
[18]/2010	Turkey	31/F	CAPD	*Candida rugosa*	Amphotericin B	Yes	Survived
[19]/2011	Taiwan	Neonate/F	Acute PD	*Candida parapsilosis*	Amphotericin B + fluconazole	Yes	Survived
[20]/2014	India	68/F	CAPD	*Candida haemulonii*	Voriconazole	Yes	Death
[21]/2015	Turkey	42/M	CAPD	*Candida guilliermondii*	Fluconazole	Yes	Survived
[22]/2016	USA	57/F	CAPD	*Candida tropicalis*	Fluconazole	Yes	Survived
[23]/2016	South Korea	53/M	CAPD	*Candida guilliermondii*	Fluconazole	Yes	Survived
[24]/2017	Greece	30/M	CAPD	*Candida parapsilosis*	Amphotericin B + fluconazole	Yes	Survived
[25]/2018	USA	74/F	CAPD	*Candida glabrata*	Fluconazole → micafungin	Yes	Survived
[26]/2018	China	55/F	CAPD	*Candida krusei*	Fluconazole → caspofungin	Yes	Survived
[27]/2021	Turkey	48/M	CAPD	*Candida pelliculosa* (*W. anomalus*)	Fluconazole + anidulafungin	Yes	Survived
[28]/2021	Mexico	22/F	CAPD	*Candida tropicalis*	Fluconazole	Yes	Survived
[29]/2021	USA	75/F	CAPD	*Candida parapsilosis*	Fluconazole	Yes	Survived
[30]/2021	Turkey	22/F	APD	*Candida guilliermondii*	Fluconazole	Yes	Survived
[31]/2023	Thailand	68/F	CAPD	*Candida nivariensis*	Fluconazole	Yes	Death
[32]/2023	Italy	84/F	CAPD	*Candida guilliermondii*	Voriconazole	Yes	Survived
[33]/2024	China	69/M	CAPD	*Candida parapsilosis*	Voriconazole	Yes	Survived
[34]/2025	USA	66/F	CAPD	*Candida glabrata*	Anidulafungin	Yes	Death

NAC, non-albicans *Candida*; PD, peritoneal dialysis; APD, automated peritoneal dialysis; CAPD, continuous ambulatory peritoneal dialysis; F, female; M, male; *W. anomalus*, *Wickerhamomyces anomalus*.

**Table 2:** Case series reporting NAC peritonitis in patients receiving PD.

Reference/Year	Country	Study Design	Episodes (*n*)	Species Distribution	Mean Age (Years)	PD Modality	Prior Antibiotics (%)	Catheter Removal (%)	Mortality (*n*)
[35]/1992	Hong Kong	Case series	5	*Candida tropicalis*	51.2	Intermittent PD	20	100	3
[6]/2000	Hong Kong	Case series	7	*Candida parapsilosis*	62 ± 11.5	CAPD	71	100	1
[7]/2002	Thailand	Case series	24 (23 patients)	*Candida parapsilosis*	52 ± 13	CAPD	23.5	100	4
[36]/2006	Taiwan	Retrospective cohort	16	*C. parapsilosis* (9), *C. tropicalis* (4), *C. famata* (3),	53.9 ± 13.7	CAPD	44	100	1
[8]/2012	Canada	Case series	8	*C. tropicalis* (3), *C. parapsilosis* (3), *C. krusei* (1), *C. glabrata* (1)	61.6 ± 13.3	PD	67	100	0
[37]/2022	Mexico	Case series	4	*C. parapsilosis* (2), *C. tropicalis* (1), *C. stellatoidea* (1)	42.8 ± 16.2	CAPD/APD	100	100	0

NAC, non-albicans *Candida*; PD, peritoneal dialysis; CAPD, continuous ambulatory peritoneal dialysis; APD, automated peritoneal dialysis.

**Table 3:** Management and outcomes of reported cases.

Variable	Case Reports	Case Series
Initial antifungal therapy		
Fluconazole (%)	60	72
Amphotericin B (%)	28	8
Echinocandins (%)	12	4
Catheter removal (%)	92	100
Switch to HD (%)	84	100
Mortality (%)	20	24

**Table 4:** Species distribution of NAC peritonitis across all included studies.

Species	Case Reports (*n*)	Case Series (*n*)	Total
*Candida parapsilosis*	5	45	50
*Candida tropicalis*	2	13	15
*Candida glabrata*	3	1	4
*Candida guilliermondii*	4	0	4
*Candida fumata*	0	3	3
*Candida lusitaniae*	2	0	2
*Candida famata*	2	0	2
*Candida krusei*	1	1	2
*Candida haemulonii*	1	0	1
*Candida sake*	1	0	1
*Candida rugosa*	1	0	1
*Candida nivariensis*	1	0	1
*Candida pelliculosa* (*Wickerhamomyces anomalus*)	1	0	1
*Candida subhashii*	1	0	1
*Candida stellatoidea*	0	1	1

NAC, non-albicans *Candida*.

**Table 5:** Clinical patterns and antifungal susceptibility signals according to the five most frequent NAC species.

Species	Total Isolates *	Prior Antibiotics (%)	Polymicrobial Infection (%)	Initial Fluconazole (%)	Catheter Removal (%)	Mortality (%)	Antifungal Susceptibility Pattern
*Candida parapsilosis*	5 (50)	80	0	80	100	20	Generally, fluconazole-susceptible; favorable clinical outcomes
*Candida tropicalis*	2 (15)	50	50	100	100	0	Usually susceptible to azoles; no resistance signal in reported cases
*Candida glabrata*	3 (4)	100	33	67	100	33	Reduced azole susceptibility: fluconazole failure or escalation to echinocandin therapy reported
*Candida guilliermondii*	4 (4)	75	25	75	100	0	Generally, azole-susceptible with good outcomes in reported cases
*Candida famata*	2 (3)	100	0	100	50	100	Possible treatment failure despite fluconazole susceptibility reported in vitro

Percentages were calculated from individual case reports with available data. Antifungal susceptibility patterns were inferred from microbiological descriptions and reported treatment responses in the original publications and should be interpreted with caution due to the small number of cases. * Numbers outside parentheses represent isolates from individual case reports, whereas numbers in parentheses indicate the total number of reported isolates across all included studies. NAC, non-albicans *Candida*.

## Data Availability

The data supporting the reported results are from previously published studies. A list of all included studies is provided in the manuscript’s reference section. The original data presented in the study are openly available in all the academic databases mentioned in the method section.

## Supplementary Materials

The following supporting information can be downloaded at https://www.mdpi.com/article/10.3390/idr18030041/s1. Supplementary Table S1: Risk-of-bias assessment of included case reports; Supplementary Table S2: Risk-of-bias assessment of included case series.

## Abbreviations

The following abbreviations are used in this manuscript:

PD	Peritoneal Dialysis
NAC	Non-albicans *Candida*
APD	Automated Peritoneal Dialysis
CAPD	Continuous Ambulatory Peritoneal Dialysis
HD	Hemodialysis
ISPD	International Society for Peritoneal Dialysis
MALDI-TOF MS	Matrix-Assisted Laser Desorption/Ionization Time-of-Flight Mass Spectrometry
PRISMA	Preferred Reporting Items for Systematic Reviews and Meta-Analyses
PROSPERO	International Prospective Register of Systematic Reviews
