# Supplementary material for: Non-Albicans Candida Peritonitis in Peritoneal Dialysis: Species Distribution, Management, and Outcomes—A Systematic Case-Based Review
Source: Infect Dis Rep. 2026 Apr 27;18(3):41. doi: 10.3390/idr18030041 (PMC13214705; doi:10.3390/idr18030041)
Supplement: Supplementary file 1 [file idr-18-00041-s001.zip › Supplementary Table S1.pdf]

Supplementary Table S1. Risk-of-bias assessment of included case reports using the Joanna Briggs Institute (JBI) critical appraisal tools.

Study (Author, Year)	Study Type	Patient Description	Diagnostic Ascertainment	Clinical Data Completeness	Outcome Reporting	Overall Quality
Quindos, 1994	Case report	Clear	Adequate	Complete	Adequate	Moderate
Maruyama, 1997	Case report	Clear	Adequate	Complete	Adequate	Moderate
Asim, 1999	Case report	Clear	Adequate	Complete	Adequate	Moderate
Cinar, 2002	Case report	Clear	Adequate	Complete	Adequate	Moderate
Tarif, 2004	Case report	Clear	Adequate	Incomplete	Adequate	Moderate
Gupta, 2006	Case report	Clear	Adequate	Complete	Adequate	Moderate
Adam, 2009	Case report	Clear	Adequate	Complete	Adequate	High
Guclu, 2009	Case report	Clear	Adequate	Complete	Adequate	Moderate
Kocyigit, 2010	Case report	Clear	Adequate	Complete	Adequate	High
Tatli, 2010	Case report	Clear	Adequate	Complete	Adequate	Moderate
Cheng, 2011	Case report	Clear	Adequate	Complete	Adequate	High
Yuvaraj, 2014	Case report	Clear	Adequate	Complete	Adequate	Moderate
Gayen, 2016	Case report	Clear	Adequate	Complete	Adequate	Moderate
Kim, 2016	Case report	Clear	Adequate	Complete	Adequate	Moderate
Balafa, 2017	Case report	Clear	Adequate	Complete	Adequate	Moderate
Emami, 2018	Case report	Clear	Adequate	Complete	Adequate	Moderate
Tong, 2018	Case report	Clear	Adequate	Complete	Adequate	Moderate
Evren, 2021	Case report	Clear	Adequate	Complete	Adequate	Moderate
Ramirez, 2021	Case report	Clear	Adequate	Complete	Adequate	Moderate
Roy, 2021	Case report	Clear	Adequate	Complete	Adequate	Moderate
Sadioglou, 2021	Case report	Clear	Adequate	Complete	Adequate	Moderate
Chamroensakchai, 2023	Case report	Clear	Adequate	Complete	Adequate	Moderate
Mastroianni, 2023	Case report	Clear	Adequate	Complete	Adequate	Moderate
Zhang, 2024	Case report	Clear	Adequate	Complete	Adequate	Moderate
Yadav, 2025	Case report	Clear	Adequate	Incomplete	Adequate	Moderate
