# Supplementary material for: Non-Albicans Candida Peritonitis in Peritoneal Dialysis: Species Distribution, Management, and Outcomes—A Systematic Case-Based Review
Source: Infect Dis Rep. 2026 Apr 27;18(3):41. doi: 10.3390/idr18030041 (PMC13214705; doi:10.3390/idr18030041)
Supplement: Supplementary file 1 [file idr-18-00041-s001.zip › Supplementary Table S2.pdf]

Supplementary Table S2. Risk-of-bias assessment of included case series using the Joanna Briggs Institute (JBI) critical appraisal tools

Study (Author, Year)	Study Type	Patient Description	Diagnostic Ascertainment	Clinical Data Completeness	Outcome Reporting	Overall Quality
Yuen, 1992	Case series	Partly clear	Adequate	Incomplete	Adequate	Moderate
Wong, 2000	Case series	Clear	Adequate	Partly complete	Adequate	Moderate
Kaitwatcharachai, 2002	Case series	Partly clear	Adequate	Incomplete	Adequate	Moderate
Chen, 2006	Case series	Partly clear	Adequate	Partly complete	Adequate	Moderate
Levallois, 2012	Case series	Partly clear	Adequate	Partly complete	Adequate	Moderate
Villa, 2022	Case series	Partly clear	Adequate	Incomplete	Adequate	Moderate
